# Assessment of dust exposure and chronic respiratory symptoms among workers in medium scale woodwork factories in Ethiopia; a cross sectional study

**DOI:** 10.1186/s12889-021-10357-z

**Published:** 2021-02-06

**Authors:** Tegegnework Yitayew Awoke, Abera Kumie Takele, Worku Tefera Mekonnen, Samson Wakuma Abaya, Yifokire Tefera Zele, Embay Amare Alemseged, Bezayit Girma Abay

**Affiliations:** 1 Addis Ababa City Administration Food, Medicine and Healthcare Administration and Control Authority, Addis Ababa, Ethiopia; 2 Department of Preventive Medicine, School of Public Health, Addis Ababa University, Addis Ababa, Ethiopia; 3 Department of Public Health, School of Medicine and Health Sciences, Adigrat University, Adigrat, Ethiopia

**Keywords:** Occupational exposure, Total dust, Chronic respiratory symptoms, Wood dust, Woodworkers, Ethiopia

## Abstract

**Background:**

Wood dust in a form of inhalable particulates can penetrate the lung tissues and affect respiratory health. Woodwork factory workers are at a greater risk of developing respiratory health problems because of exposure in their working environment, but existing data were few. The aim of this study was to assess the prevalence of chronic respiratory symptoms, associated factors, and concentration of personal total wood dust level among medium-scale woodwork factory workers.

**Methods:**

An institutional based cross-sectional study was conducted among 506 woodwork factory workers. We selected study participants using a simple random sampling technique. We assessed chronic respiratory symptoms using the British Medical Research Council respiratory symptoms questionnaire with a few modifications. A multivariate logistic regression model was used to identify the factors. Forty dust measurements were collected from 20 randomly selected workers using a closed-face cassette (CFC) personal sampler. We analyzed the dust samples gravimetrically using a standard microbalance scale.

**Results:**

We recruited a random sample of 506 workers in the study with a response rate of 98%. The prevalence of chronic respiratory health symptoms among woodworkers was 69.8% with a prevalence of cough (54.6%), phlegm (52.2%), wheezing (44.6%), breathlessness (42.1%), and chest pain (42.9%). Past occupational dust exposure history (AOR = 2.09, 95% CI; 1.09–4.01), work experience > 5 years (AOR = 9.18, 95% CI; 5.27–16.00), using bio-fuel as energy for cooking (AOR = 2.42, 95% CI; 1.44–4.07), and having no occupational safety and health training (AOR = 3.38, 95% CI; 1.20–9.49) were factors that significantly associated with chronic respiratory symptoms among woodwork workers. The geometric mean (GM) of dust exposure level among woodworkers was 10.27 mg/m^3^, which exceeded the limit of 10 mg/m^3^ set by the ACGIH.

**Conclusions:**

High prevalence of chronic respiratory symptoms was reported from woodwork factory workers. Increased work- experience, using bio-fuel as an energy source for cooking, past occupational dust exposure history, and having no occupational safety and health training were identified risk factors. The measured average personal wood dust exposure level was above the recommended occupational threshold limit value. Therefore, workers’ wood dust exposure reduction and control methods and respiratory health awareness programs should be implemented.

## Background

Occupational respiratory diseases are major global public health problems that account for up to 30% of all registered work-related diseases with up to 50% prevalence among workers in high-risk sectors such as mining, construction, and dust-generating works [1]. All countries of the world have experienced serious public health problems due to chronic respiratory diseases [2]. According to Fedotov (2011), deaths from respiratory diseases accounted for 8% from annual work-related deaths [1]. Among 2.77 million work-related fatalities in 2015, 17.1% accounts for respiratory diseases [3]. World Health Organization (WHO) reported that deaths from respiratory diseases in Ethiopia were 4% in 2011 and 3% in 2014 [4, 5].

Occupational diseases such as respiratory symptoms are the major risks for woodworkers**.** Chronic respiratory health symptom is one of the major health problems of woodworkers resulting from breathing in noxious or toxic chemicals such as wood dust [6, 7]. Wood dust, which is an organic dust, is one of the most occupational exposure woodworkers are exposed during work causing several respiratory diseases [8]. Wood dust is classified as a human carcinogen by the International Agency for Research on Cancer [9, 10].

Higher prevalence of chronic respiratory symptoms was found among wood workers in Ethiopia, [11–13]. Previous studies conducted in Ethiopia investigated the prevalence of chronic respiratory symptoms among woodworkers [11–13] and personal wood dust concentration level of workers in particleboard and small-scale woodwork enterprises [13, 14]. They did not investigate, however, the prevalence of chronic respiratory symptoms, factors associated with chronic respiratory symptoms, and concentration of personal total dust exposure level among workers in medium-scale woodwork factories together in Ethiopian context. Therefore, this study aimed to assess prevalence of chronic respiratory health symptoms, to identify factors associated with chronic respiratory health symptoms, and estimate personal total wood dust exposure levels among workers in medium-scale woodwork factories in Addis Ababa, Ethiopia.

## Methods

### Study area, period, and design

The study area is Akaki-Kality sub-city of Addis Ababa, Ethiopia as the great majority of woodwork factories are found in this area. With a population of 3,604,000 as of 2019 [15], Addis Ababa is the world’s largest city that is in a landlocked country. Currently, the city has 10 sub-cities with 116 woredas [16]. Among the 10 sub-cities of Addis Ababa, Akaki-Kality sub-city is the largest sub-city with 13 woredas. The sub-city has a total area of 118.08 km^2^ [17] with a population size of 227,182; 110,435 males and 116,747 females [18]. An institutional based cross-sectional study was conducted among woodwork factory workers from 14 March 2019 to 22 May 2019 in Akaki Kality Sub City, Addis Ababa, Ethiopia.

### Source and study population

All medium-scale woodwork factories and workers engaged in these factories in Akaki-Kality sub-city, Addis Ababa was the source population. The study population was selected medium-scale woodwork factories and workers engaged in the sawing and sanding activities and those who had worked for more than 1 year in these factories in Akaki-Kality sub-city of Addis Ababa, Ethiopia.

### Selection criteria

All workers above 18 years of age who had direct involvement in the sawing and sanding area were included. Workers who had worked in woodwork activities for 1 year and above in medium scale woodwork factories were included in the study. On the other hand, those who had heart failure, recent surgery of thorax, abdomen, and any acute illness before employed as woodworker were excluded from the study.

### Sample size determination

The sample size was determined using double population proportion formula using Epi Info Version 7.2 software considering the following assumptions: P_1_: 60.4% of woodworkers who worked for 10 years and above developed at least one chronic respiratory symptoms [19] with Odds Ratio (OR) = 1.86, 95% confidence interval, 85% power, 5% margin of error, 1:1 ratio of exposed to unexposed. The sample size became 460 (*n* = 460). Adding 10% for non-response rate, 506 study participants were included in this study.

### Sample size for exposure assessment

For personal wood dust samples, the sample size was determined based on Rappaport and Kupper’s recommendations for exposure studies. According to Rappaport and Kupper, 5 to 10 randomly selected individuals with 10 to 20 measurements of exposure per similar exposure groups (SEGs) are adequate to estimate the exposure level for dust sampling [20]. Two working activities, i.e. sawing and sanding, constituted two SEGs and 10 randomly selected workers for sawing and 10 randomly selected workers for sanding, a total of 20 randomly selected workers with repeated measurement constituted the sample. Forty dust measurements, 20 from sawing and 20 from sanding, were undertaken for dust measurement as this amount of sample is believed to be adequate to estimate the exposure level in SEGs [20].

### Sampling technique

As part of the interview, we randomly selected 12 medium-scale woodwork factories from 40 medium-scale woodwork factories found in Akaki Kality sub city. We proportionally allocated the samples from each factory. We used the workers’ roster as a sampling frame. The selection of study participants was done by a simple random sampling method.

### Data collection procedure

#### Interviews

A modified version of the British Medical Research Council (BMRC) questionnaire [21, 22] with few modifications was used to assess the prevalence of chronic respiratory symptoms and factors associated with chronic respiratory symptoms. The components of the questionnaires were sociodemographic variables, work-related variables, common chronic respiratory symptoms variables, behavioral factors of workers, type of fuel used for cooking, and previous history of respiratory illnesses. Trained data collectors performed face-to-face interviews in Amharic. Five Environmental Health Professionals with B.Sc. degree were engaged in the interview as a data collector and one senior Environmental Health Professional with a B.Sc. degree was engaged as a supervisor. Two days of training was given for data collectors on the questionnaire and method of the interview as well as observational checklist.

An observational checklist was also used to assess the availability and utilization of respiratory protective equipment (RPE) and working environment (ventilation, and dust accumulation).

#### Dust measurement

Personal total dust was sampled using pre-weighed polyvinyl chloride (PVC) filter membranes with a pore size of 5.0 μm placed on a Millipore plastic closed face 37 mm filter cassette (CFC) connected to Side Kick Casella (SKC) pump through which air was pumped by a rechargeable battery-powered motor at a constant flow rate of 2.0 l/min [23]. The sampling cassettes are situated on shoulder straps as close to the breathing zone (30 cm from nostril) as possible. Samplers were adjusted in front of either the right or the left shoulder randomly to reduce bias that came from their position.

Wood dust samples from workers were taken from 9 a.m. to 12 p.m. to make sure that the sampling did not result in an overload of the filters. This sampling duration agrees with the National Institute for Occupational Safety and Health (NIOSH) manual that considers using either full time or a portion of time with the assumption that exposure was similar [24]. The dust samples were analyzed gravimetrically using a standard microbalance scale.

### Data analysis

Collected data were organized and entered Epi info version 7.2 and we did cleaning to avoid missing values, outliers, and other inconsistencies. For data cleaning, frequency, sort, and list were used. Cleaned data exported to SPSS version 21 for analysis. Descriptive statistics were used to summarize data. Logistic regression analysis was used to identify whether exposure variables are significantly associated with outcome variables or not. Thus, variables with *p* < 0.2 were included in the multivariable analysis by adjusting confounding variables; past occupational dust exposure history, work experience, energy used for cooking, OSH training. We considered variables as significant independent factors based on Adjusted Odds Ratio (AOR) with 95% CI and *P*-value of < 0.05. The P-value of < 0.05 was considered as statistically significant.

The dust samples were analyzed gravimetrically using a standard microbalance scale. Exposure data were recorded in an excel spreadsheet and exported into SPSS Version 21 for descriptive analysis. The result of the dust concentration was described using descriptive statistics such as measures of central tendency (Geometric Mean; GM) and a measure of dispersion (Geometric Standard Deviation; GSD). We used one-way Analysis of variance (ANOVA) to compare dust concentration variation between and within groups (tasks or departments). The dust level was described and compared with the threshold limit value (TLV) of the American Conference of Governmental Industrial Hygienists; ACGIH recommendation (10 mg/m^3^).

### Data quality assurance

**For the interview,** a standardized questionnaire modified from the British Medical Research Council (BMRC) [21, 22] was used to assure data quality. Before data collection, 2 days of training were given to the data collectors to fill the questionnaire appropriately and to reduce bias. Additionally, the questionnaires were first translated from English to Amharic and back-translated to English using standard procedure to check its validity. Each data collector has checked the questionnaires frequently for completeness and consistency. At the end of the day, we checked all the filled questionnaires. Investigators cautiously observed and followed the overall activities of the study. To improve recall, questions on the presence of symptoms were limited to occur within 3 months before the conduct of the study.

For dust sampling, any weight changes due to atmospheric conditions and handling of the samples during transportation and sampling were corrected using field blanks. We capped and transported the sampling head to the laboratory for post weighing after sampling was completed. Polyvinylchloride (PVC) filters stayed in the cassettes until weighing was made at the laboratory, which required 24 h of conditioning in desiccators. We used the same way transportation mechanism to transport the samples and blanks to the laboratory in a labeled suitable container to prevent any damage and disturbances. We followed standard operative procedures (SOPs) to ensure the quality of weighing using the microbalance. Field supervisors checked the position of the sampling head every 30 min and consistently observed the study participants throughout the sampling period.

Pilot study or pre-testing was carried out a week before the actual data collection in Kirkos sub-city, Addis Ababa to check the competency of the data collectors, and the reliability and validity of the data collection tools and necessary corrections were taken accordingly.

### Variable measurement

#### Outcome variables

Chronic respiratory symptoms: are defined as the development of one or more of the respiratory symptom(s) of a chronic cough, chronic cough with sputum, chronic breathlessness, chronic wheezing, chronic chest illness which lasts at least 3 months in 1 year [25].

#### Exposure variables

The socio-demographic factors: age (< 30 years, ≥ 30 years), sex (male, female), marital status (single, married, divorced, widowed), educational status (illiterate, primary education, secondary education, certificate and above), and monthly income (≤ 2500 ETB and >  2500 ETB) were included.

Behavioral factors of workers: smoking habit (current smokers:-workers who were smoking at the time of the study or a person who smokes cigarettes every day or some days [26]. The work-related factors include work experience (1–5 years, > 5 years); working hours per week (> 40 h, ≤ 40 h), working department (sawing, sanding); previous dust exposure history: - workers experience in the dusty environment before the current working position [27].

Previous respiratory illnesses (like the history of asthma, chronic bronchitis, lung cancer and tuberculosis, emphysema, pneumonia) that could be developed before and confirmed by physicians, energy used for cooking: bio-fuel (including wood, coal, and gas) and electricity as well as occupational safety and health training and RPE utilization.

Dust: consisting of particles in the environment that originates from several sources such as wood dust [28].

### Ethical consideration

The study was conducted after having an ethical clearance from the Institutional Review Board of the College of Health Sciences of Addis Ababa University. Before performing measurements, verbal and written consent was also obtained from each study participants and we informed them that they have full rights to refuse and discontinue taking part at any point in the study. The study participants with developed chronic respiratory health symptoms were advised and linked to a health facility.

## Results

### Socio-demographic characteristics

Five hundred six (506) woodworkers from 12 medium scale woodwork factories were included to participate in this study, of which 496 participated with a response rate of 98.02%. Ten individuals were unwilling to participate and excluded from the study. Twenty workers were involved in dust measurement.

Most study participants were males, who account for 84.5% with the median age of 30 years and 253 (51.0%) were at the age of 30 years and above. Two hundred thirty-nine (48.2%) respondents were married and 232 (46.8%) of the respondents attended secondary education. The median income of the respondents was 2500.00 ETB **(**Table 1**).**

**Table 1:** Socio-demographic characteristics of workers in medium-scale woodwork factory in Addis Ababa, Ethiopia, 2019

Variable		Frequency (***n*** = 496)	Percentage
**Sex**	Male	419	84.5
	Female	77	15.5
**Age in years**	< 30	243	49.0
	≥ 30	253	51.0
**Marital Status**	Single	207	41.7
	Married	239	48.2
	Divorced	35	7.1
	Widowed	15	3.0
**Educational Status**	Illiterate	21	4.2
	Primary Education	192	38.7
	Secondary Education	232	46.8
	Certificate and above	51	10.3
**Monthly Income in ETB**	≤ 2500	286	57.7
	> 2500	210	42.3

### Workplace characteristics and Behavioral factors

Hundred thirty (26.2%) of woodwork factory workers had an experience of dust exposure before joining the present job. From woodwork factory workers, 55.4% had work experience in the factory for 5 years or more. Eighty-eight (17.7%) of woodwork factory workers were working more than forty hours (> 40 h) per week. Sixty-two (12.5%) woodwork factory workers were reported chronic respiratory disease confirmed by physicians before they have started a job in the factory. In this study, 69 (13.9%) of woodwork factory workers had a history of cigarette smoking behavior. A nearly an equal number of respondents worked in sanding (49.2%) and sawing (50.8%) departments. None of the respondents used a proper type of RPEs. One hundred fifty-three (30.8%) used pieces of clothes as respiratory protective equipment. Only 5 % of respondents had training on occupational safety and health (Table 2).

**Table 2:** Workplace characteristics and behavioral factors of workers in medium-scale woodwork factory, Addis Ababa, Ethiopia, 2019

Variables		Frequency (n = 496)	Percentage
**Working Department**	Sawing	247	49.8
	Sanding	249	50.2
**Work experience**	> 5 years	275	55.4
	1–5 years	221	44.6
**Working hours per week**	> 40 h	88	17.7
	≤ 40 h	408	82.3
**Past Occupational dust exposure history**	Yes	130	26.2
	No	366	73.8
**Previous respiratory illness**	Yes	62	12.5
	No	434	87.5
**Energy used for cooking**	Bio-fuel	210	42.3
	Electricity	286	57.7
**Cigarette smoking**	Yes	69	13.9
	No	427	86.1
**Occupational Safety and Health Training**	Yes	25	5.0
	No	471	95.0
**Used pieces of cloth as RPE**	Yes	153	30.8
	No	343	69.2

### Prevalence of chronic respiratory symptoms

**Fig. 1:**
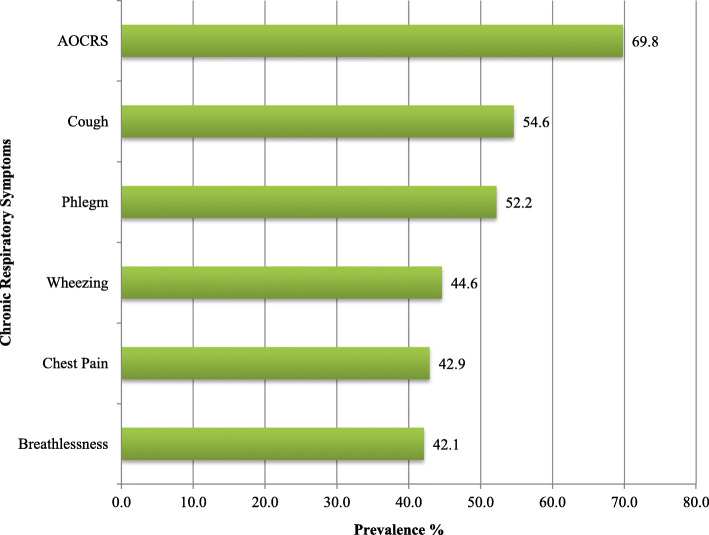
Prevalence of chronic respiratory symptoms in medium-scale woodwork factory workers, Addis Ababa, Ethiopia, 2019 (n = 496)

Three hundred forty-six (69.8%) [95% CI: 66.0–73.8] of woodworkers ever had at least one chronic respiratory symptoms (AOCRS). The prevalence’s of cough, phlegm, wheezing, chest pain, and breathlessness with 95% confidence interval among woodwork factory workers were 54.6% CI (50.8–58.9), 52.2% CI (48.0–56.6), 44.6% CI (40.1–49.1), 42.9% CI (39.1–47.2) and 42.1% CI (38.1–46.7), respectively (Fig. 1).

### Previous history of respiratory illnesses

**Fig. 2:**
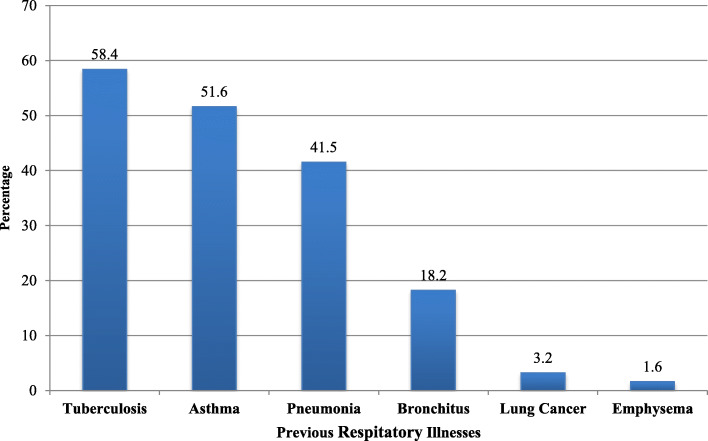
Previous history of respiratory illnesses in medium-scale woodwork factory workers, Addis Ababa, Ethiopia, 2019 (*n* = 62)

Sixty-two (12.5%) of woodwork factory workers had a previous history of chronic respiratory diseases confirmed by physicians before they have started woodworking. From these workers, 10 (18.2%) had chronic bronchitis, 1(1.6%) had emphysema, 32 (51.6%) had asthma, 2 (3.2%) had lung cancer, 34 (54.8%) had tuberculosis (TB) and 26 (41.9%) had pneumonia (Fig. 2).

### Workplace observation

Observational findings of this study showed that wood dusts were accumulated on the walls and floors of working sections of the factories due to lack of dust absorber, artificial, and natural ventilation. Regarding respiratory protective equipment usage, the organizations did not provide a proper type of respiratory protective equipment (RPE) for workers. None of the workers used a proper type of RPEs. They rather used pieces of cloth as RPEs.

#### Factors associated with chronic respiratory symptoms

The multivariate analysis was summarized in Table 3. Past occupational dust exposure history, work experience, energy used for cooking, and occupational safety and health training of the workers were significantly associated with chronic respiratory symptoms (*p* < 0.05).

**Table 3:** Chronic Respiratory Symptoms and associated factors in medium-scale woodwork factories workers, Addis Ababa, 2019

Variables	At least one chronic respiratory symptom		COR (95%CI)	AOR (95%CI)
	Yes	No		
**Age in Years**				
< 30	130	113	1.00	1.00
≥ 30	216	37	5.07 (3.30–7.80)*	1.55 (0.90–2.66)
**Marital Status**				
Single	120	87	1.21 (0.42–3.45)	1.60 (0.51–5.04)
Married	192	47	3.57 (1.23–10.35)*	2.75 (0.88–8.63)
Divorced	26	9	2.53 (0.71–8.97)	1.98 (0.51–7.53)
Widowed	8	7	1.00	1.00
**Monthly income in ETB**				
≤ 2500	190	96	0.68 (0.46–1.08)*	1.09 (0.69–1.71)
> 2500	156	54	1.00	1.00
**Past Occupational dust exposure history**				
Yes	113	17	3.79 (2.18–6.59)*	2.09 (1.09–4.01)* *
No	233	133	1.00	1.00
**Previous respiratory illnesses**				
Yes	56	6	4.63 (1.95–11.01)*	2.05 (0.74–5.66)
No	290	144	1.00	1.00
**Work experience**				
> 5 years	250	25	13.02 (7.98–21.25)*	9.18 (5.27–16.00)* *
1–5 years	96	125	1.00	1.00
**Working hours per week**				
> 48 h	71	17	2.02 (1.14–3.57)*	0.85 (0.41–1.78)
≤ 48 h	275	133	1.00	1.00
**Working Department**				
Sawing	175	72	1.11 (0.76–1.63)	0.83 (0.51–1.33)
Sanding	171	78	1.00	1.00
**Energy used for Cooking**				
Bio-fuel	178	32	3.91 (2.51–6.09)*	2.42 (1.44–4.07)* *
Electricity	168	118	1.00	1.00
**OSH Training**				
Yes	13	12	1.00	1.00
No	333	138	2.23 (0.99–5.00)*	3.38 (1.20–9.49)* *
**Cigarette Smoking**				
Yes	64	5	6.58 (2.59–16.71)*	2.11 (0.73–6.09)
No	282	145	1.00	1.00

1.00 = reference value, *P < 0.2 for COR, **P < 0.05 for AOR, *COR* crude odds ratio, *AOR* adjusted odds ratio

The odds of chronic respiratory health symptoms among woodworkers who had past occupational dust exposure history was 2.09 times that of workers who did not have previous dust exposure (AOR = 2.09, 95% CI; 1.09–4.01). The odds of chronic respiratory health symptoms among workers who had work experience above 5 years (> 5 years) was 9.18 times that of workers who had work experience between one to 5 years (AOR = 9.18, 95% CI; 5.27–16.00). The odds of chronic respiratory health symptoms among workers who used bio-fuel for cooking was 2.42 times that of workers who used electricity for cooking (AOR = 2.42, 95% CI; 1.44–4.07). Having no occupational safety and health training (OSH training) was significantly associated with chronic respiratory health symptoms among woodworkers. The odds of chronic respiratory health symptoms among workers who had no occupational safety and health training was 3.38 times that of workers who had occupational safety and health training (AOR = 3.38, 95% CI; 1.20–9.49).

### Personal wood dust exposure

The geometric mean (GM) ± geometric standard deviation (GSD) of the overall total personal wood dust concentration was 10.27 (± 1.71) with a range varying from 4.28–26.24 mg/m^3^. Out of the total 40 dust samples, 22 (55%) of them were above 10 mg/m^3^ of the American Conference of Industrial Hygienist (ACGIH) recommendation. Comparing dust exposure concentration with task operation, there was no significant difference between sawing and sanding tasks (df = 1, F = 0.098, *p* = 0.756).

#### Measurement of wood dust exposure in the sawing department

In total, 20 samples from 10 workers involved in the sawing activity with two-repeated measurements were collected and the level of exposure ranged from 4.30 to 26.24 mg/m^3^ with a GM of 10.40 mg/m^3^ and GSD of 1.70. In this department, 59.1% (*n* = 13) of the measurements were above the TLV set by the ACGHI.

#### Measurement of wood dust exposure in the sanding department

Twenty samples were collected from 10 workers involved in the sanding activity with two-repeated measurements. Personal exposure to wood dust among workers varied between 4.28 and 23.67 mg/m^3^ with a geometric mean (GM) of 10.14 mg/m^3^ and a geometric standard deviation (GSD) of 1.68. During the whole sampling period, 45% (*n* = 9) of the measurements from sanding activity were above the TLV set by the ACGHI.

## Discussion

The finding of this study revealed that the overall prevalence of chronic respiratory health symptoms among woodwork factory workers was 69.8% (95% CI: 66.0–73.8). This is consistent with a study conducted (68%) among wood workers in South-South Nigeria [19] and comparable to what was obtained (62%) among woodworkers in Jos, Nigeria [29] but relatively higher than the study conducted in North East of Thailand in which only 29.9% of woodworkers had at least one chronic respiratory health symptoms [7]. This difference could be due to the variation in the use of a proper type of respiratory protective equipment (RPE). No one used any proper type of respiratory protective equipment (RPE), and only (30.8%) workers used cloth masks as RPE in this study. The observed difference could also be explained by the study setting in which the study was done, and the large sample size of the current study. The result of this study is relatively lower than the study conducted in Benin City, Nigeria in which 87.3% of woodworkers had at least one respiratory symptom [30]. This difference could be due to the variation in the selection of study participants in which 42.7% of workers were smokers [30]. Only 13.9% of workers were smokers in the present study. This study also found that a high prevalence of cough (54.6%), phlegm (52.2%), wheezing (44.6%), chest pain (42.9%), and breathlessness (42.1%). This might be due to the high concentration of wood dust found in the working area. This is supported by the personal sampling of the study sites that showed concentrations of total wood dust clearly over TLV set by ACGIH [31] that obtained from working sites with a clear absence of any form of dust control mechanism in place and the proper type of RPE. This finding is comparatively higher than the study done in Southwest Ethiopia, Jimma town with cough (41.4%), phlegm (34.3%), wheezing (12.4%), chest pain (32.9%) and breathlessness (21.5%) [11], a study done in Northeast of Thailand with cough (18.8%), phlegm (15.7%), wheezing (5.9%), and breathlessness (7.8%) [7] and a study done in Ethiopia with cough (39%), phlegm (27%), wheezing (45%), and breathlessness (24%) [13]. The difference might be due to a higher percentage (24.1%) of workers were wearing masks while working and workers had a high level of knowledge and attitude toward dust prevention in the previous studies [7]. This difference could also be due to the high concentration of wood dust found in the working area, absence of dust control mechanisms, and absence of proper type of RPE in the present study. The high prevalence of chronic respiratory health symptoms found in this study is also higher than the prevalence of chronic respiratory symptoms at a community level in Ethiopia [32]. The high prevalence of chronic respiratory health symptoms found in the present study might also be due to a longer duration of exposure to wood dust and lack of occupational safety and health training.

Past occupational dust exposure history in the woodwork factories was a major factor in the development of chronic respiratory symptoms. Having past occupational dust exposure history increased significantly woodworkers’ risk of developing chronic respiratory symptoms. This study is in line with a study conducted in Tanzania, which stated that previous dusty jobs are three times more likely to have difficulty of breathing and breathlessness as the respiratory health symptoms than free from the previous dusty job [33]. This study is also similar to a study conducted in Ethiopia, which stated that workers who had past dust exposure history were 1.86 times more likely to have at least one chronic respiratory health symptoms than workers who had no past dust exposure history [28]. The reason for the significant association of past occupational dust exposure history with the development of at least one chronic respiratory symptom might be due to misunderstanding and lack of training and knowledge on RPE utilization of workers as well as workers who had experience on dust exposure may ignore in using RPE as they consider themselves adapting the dust. However, this finding was not in line with a study conducted among woodworkers in South-South Nigeria [19]. As stated somewhere else in previous study that workers with a previous exposure ignore to use of personal protective equipment because they think they adapt to the dust. This statistical significance could be because the workers might have previously worked in dusty jobs identified to cause the respiratory problem, so this may lead to the aforementioned respiratory tissue physiologic change in later life and exacerbate the occurrence of respiratory symptoms [28].

Duration of employment or work experience in the woodwork factories was also a major factor in the development of chronic respiratory symptoms. Working for more than 5 years increased significantly one’s risk of developing chronic respiratory symptoms in this study. Previous studies reported similar findings which stated that the presence of at least one respiratory symptom among woodworkers was significantly associated with duration of work [6, 19, 34, 35]. The reason might be due to the highest accumulation of dust in the respiratory system associated with extended duration exposure at workplaces.

The finding of the present study revealed that using bio-fuel, as energy for cooking was also significantly associated with chronic respiratory symptoms among woodworkers. This result was in agreement with a previous studies conducted in Ethiopia and Nepal [32, 36]. The reason might be due to the higher risk for serious health outcomes of biofuels for cooking and heating as compared with those who use cleaner energy for cooking. Bio-fuel constituents have an irritation effect on the airways that causes them to thicken as a result of inflammation, oxidative lung damage, and protease/anti-protease imbalance which in turn leads to the development of chronic respiratory symptoms [32].

Only 5 % of woodworkers had occupational safety and health (OSH) training in this study. Lack of training on OSH has been reported in previous studies [37–39]. This finding was also comparable with a study conducted in Ethiopia among particle board workers that identified only 10% of workers attended safety and health training [40]. Lack of Occupational safety and health training was significantly associated with the development of chronic respiratory symptoms among woodworkers in the present study. Having no occupational and safety training increased significantly one’s risk of developing chronic respiratory symptoms. This result was in line with a previous study that has reported similar finding [35]. The main reason for this outcome may be that having OSH training has a greater effect in changing workers’ attitude in preventing chronic respiratory health symptoms, and provides the appropriate skills and knowledge to protect them from wood dust.

The geometric mean (GM) ± geometric standard deviation (GSD) of the overall personal wood dust concentration for the selected two woodwork factories was 10.27 (± 1.71) with a range varying from 4.28–26.40 mg/m^3^. This finding was consistent with the study conducted among workers in Tanzania [33]. But from this finding, we observed that the overall personal total dust concentration was higher than that of the previous study conducted in Tanzania [41], Ethiopia [14], and Sweden [42]. This discrepancy might be due to the seasonal variation, i.e., the data collection time in the present study was somewhat dry season hence the dust might be dispersed and lack of proper natural and artificial ventilation as well as lack of local exhaust ventilation in the woodwork factories. The medium-scale woodwork factories have a shortage of space (smaller rooms) and work with the absence of ventilation. Working rooms in most factories remained the same for the two tasks. This situation might have significantly contributed to the presence of higher wood dust concentration levels in the sanding (GM = 10.14 mg/m^3^) and sawing (GM = 10.40 mg/m^3^) departments. The geometric mean (GM) of the overall personal total wood dust concentration in this study was also exceeded the American Conference of Governmental Industrial Hygienist (ACGIH) Threshold Limit Value (TLV) recommendation of 10 mg/m^3^ [31]. This high level of dust exposure might result from the absence of natural as well as artificial ventilation. Lack of local exhaust ventilation in the factories also created a condition that generated and suspended dust around the breathing area of the workers. Shortage of space (smaller rooms) and using these smaller rooms for different tasks might have significantly contributed to the presence of higher wood dust concentration levels. No statistical difference was observed in dust exposure concentration between the departments or tasks. This was probably due to the two tasks (sawing and sanding) are performed in the same room; which was confirmed during factory observation.

### Study limitations

One of the limitations of this study is the healthy workers effect; workers who developed the symptoms may have quit the job. The use of the questionnaire method was also another limitation of this study because it may cause participants to recall bias and interviewer bias. Using 37 mm Millipore plastic sampling head (CFC) in this study significantly underestimated coarse particles in the inhalable dust fraction particles (30–100 μm). Sample losses may also occur because of the adhering of dust to the interior part of the cassette walls that leads to the underestimation of dust concentration. The best way to measure exposure to dust is by covering the entire working time. In this study, however, we assumed that the tasks were similar throughout the day and hence we considered using only half the morning shift. Only a few woodwork factories and few woodworkers were sampled for personal wood dust exposure assessment. Hence, this makes it difficult to look at the association of chronic respiratory health symptom with dust concentration. Other factors like alcohol addiction, drug addiction, and khat chewing were not included in this study, which may affect the outcome of interest.

## Conclusion

The overall prevalence of chronic respiratory health symptoms among wood workers was high. None of the respondents had a proper type of respiratory protective equipment and few woodworkers had occupational safety and health training. All the factories lack dust absorber, artificial, and natural ventilation. Work experience, energy used for cooking, past occupational dust exposure history and occupational safety, and health training were determinant factors for the occurrence of chronic respiratory symptoms. Out of the total measurement samples (*n* = 40), 55% of the woodworkers were exposed to wood dust concentration that was exceeded the occupational exposure limit recommended by the ACGIH Threshold Limit Value (TLV) guideline of 10 mg/m^3^. Effective dust control measures, provision of proper types of respiratory protective equipment (RPE), and training on occupational safety and health issues were crucial for the maintenance of respiratory health of workers engaged in woodwork factories.

## Data Availability

The datasets generated and/or analyzed during the current study is not publicly available due to study participant privacy/consent agreements but are available from the corresponding author on reasonable request.

## Abbreviations

ACGIH: American Conference of Government Industrial Hygienists
AOR: Adjusted odds ratio
BEIs: Biological exposure indices
BMRC: British Medical Research Council
CI: Confidence interval
CFC: Closed face cassette
COPD: Chronic obstructive pulmonary disease
COR: Crude odds ratio
df: degree of freedom
Epi Info: Epidemiological information package
ETB: Ethiopian birr
GM: Geometric mean
GSD: Geometric standard deviation
ILO: International Labor Organization
OR: Odds ratio
PPEs: Personal protective equipment’s
RPE: Respiratory protective equipment’s
SD: Standard deviation
SEGs: Similar exposure groups
SPSS: Statistical package for social sciences
TB: Tuberculosis
TLV: Threshold limit value
WHO: World Health Organization
